# Tailoring of Thermo-Mechanical Properties of Hybrid Composite-Metal Bonded Joints

**DOI:** 10.3390/polym13020170

**Published:** 2021-01-06

**Authors:** Tasnuva Khaleque, Xiaolong Zhang, Vijay Kumar Thakur, Adrianus Indrat Aria, Hamed Yazdani Nezhad

**Affiliations:** 1 Department of Mechanical Engineering and Aeronautics, City University of London, London EC1V 0HB, UK; Tasnuva.Khaleque@city.ac.uk; 2 Enhanced Composites and Structures Centre, Cranfield University, Cranfield MK43 0AL, UK; xiaolong.zhang@cranfield.ac.uk; 3 Biorefining and Advanced Materials Research Center, Scotland’s Rural College (SRUC), Kings Buildings, West Mains Road, Edinburgh EH9 3JG, UK; Vijay.Thakur@sruc.ac.uk; 4 Department of Mechanical Engineering, School of Engineering, Shiv Nadar University, Uttar Pradesh 201314, India; 5 Surface Engineering and Precision Centre, Cranfield University, Cranfield MK43 0AL, UK

**Keywords:** composite-metal joint, electric vehicles, carbon nanotubes, polymer adhesive, thermal strain measurement, coefficient of thermal strain mismatch

## Abstract

Metallic substrates and polymer adhesive in composite-metal joints have a relatively large coefficient of thermal expansion (CTE) mismatch, which is a barrier in the growing market of electric vehicles and their battery structures. It is reported that adding carbon nanotubes (CNTs) to the adhesive reduces the CTE of the CNT-enhanced polymer adhesive multi-material system, and therefore when used in adhesively bonded joints it would, theoretically, result in low CTE mismatch in the joint system. The current article presents the influence of two specific mass ratios of CNTs on the CTE of the enhanced polymer. It was observed that the addition of 1.0 wt% and 2.68 wt% of multi-walled CNTs (MWCNTs) decreased the CTE of the polymer adhesive from $7.5\times 10^{-5}$ °C${}^{-1}$ (pristine level) to $5.87\times 10^{-5}$ °C${}^{-1}$ and $4.43\times 10^{-5}$ °C${}^{-1}$, respectively, by 22% and 41% reductions.

## 1. Introduction

Due to their advantages of energy saving and environmental protection, high-performance electric vehicles are under rapid development [1]. The structure adhesive bonding technology, as a new type of lightweight structural joining technology nowadays, exhibits good performance when joining dissimilar materials, such as those identified in, and solves the technical bottlenecks in conventional joining processes [2,3,4,5,6,7]. Moreover, it has good sealing and fatigue resistance compared to traditional joining methods, which make them suitable for the assembling process of high-performance electric vehicles [8]. For example, the adhesive bonding technology is applied in the battery pack system, which is one of the core components of electric vehicles to determine the mileage and speed of electric vehicles [9]. The disadvantage of this battery pack system is that the batteries discharge at different rates during use, and generate significant heat at different rates of heat generation. Additionally, the accumulation of time and space effect will accumulate heat. The temperature in the battery pack area can reach up to 200 °C [10]. The high temperature may lead to significant thermal expansion and residual stress in the battery compartments, resulting in failure of composite-metal bonded joints. The joint failures in the battery pack system will result in some serious problems, such as cracks in joints and moisture in the air entering the battery [8], thus reducing the performance of the battery, and even leading to the failure of the entire battery pack system, potentially leading to traffic accidents. The main factor impacting the failure of the bonding joint is the coefficient of thermal expansion (CTE) mismatch among components of the joint, especially between the metal and polymer adhesives. CTE mismatch is the main source of inducing internal shear stress that may result in joint failure at high temperatures. It has been reported in previous studies that carbon nanotubes (CNTs) can be utilised as additives in epoxy-based adhesives to engineer their CTEs, allowing for the manufacture of enhanced adhesives with tailorable CTEs to meet the requirements of a wide range applications [11,12,13,14].

The present study investigates how the addition of varying weight percentages of CNTs in adhesive affects the CTE of the adhesive in adhesively bonded aluminium–epoxy polymer joints.

## 2. Experimental Approach

### 2.1. Materials

A low viscosity bisphenol-A epoxy resin, Araldite LY1564 (Hunstman, UK), was used in the present study. The epoxy was cured using a hardener, Aradur 3486 (Hunstman, UK). The mix ratio of Araldite LY1564 and Aradur 3486 was 100:34 by weight. The recommended working temperature of the epoxy system is up to 120 °C [15].

The epoxy was modified with varying weight percentages of multi-walled CNTs (MWCNTs). The MWCNTs were synthesised in-house by injection floating catalyst chemical vapour deposition (CVD) method at 750 °C using ferrocene as the catalyst and toluene as the carbon feedstock. Iron-containing catalysts, such as ferrocene in this study, are known to result in the formation of MWCNTs [11,16,17]. The weight percentages of MWCNTs used were 1.0 and 2.68 wt%. The density of MWCNTs is almost similar to that of polymers (1–2 gm/cm${}^{3}$). A straightforward weight percentage (wt%) calculation is provided by the equation below [18]:

(1) $$V_{CNT}={\left(\frac{\left(\frac{\rho_{CNT}}{\rho_{polymer}}\right)}{{wt}_{CNT}}-\frac{\rho_{CNT}}{\rho_{polymer}}+1\right)}^{-1}$$

This implies that taking densities for the epoxy and MWCNTs at 1.25 and 1.50 gm/cm${}^{3}$, respectively, the MWCNT volume fraction will be at 0.08% and 0.42% for 0.1 and 0.5 wt%, respectively. For MWCNTs with a diameter of 15–57 nm and a length of 600 μm, it has been possible to reduce the CTE of the polymer from $7\times 10^{-5}$ °C${}^{-1}$ to $0.4\times 10^{-5}$ °C${}^{-1}$ using 8.5 vol% of MWCNTs [11]. Metals such as steel and aluminium have a CTE in the range of $1-2\times 10^{-5}$ °C${}^{-1}$. Thus, assuming a linear relation, MWCNTs with 6 vol% should reduce the CTE of the polymer to the range of steel or aluminium (theoretically). The MWCNTs used in the present study have diameters of 100–200 nm (four times radially larger than the MWCNTs used in a previous study [11]). Therefore, theoretically, to reduce the usage of costly MWCNTs up to four times to produce 6 vol% according to [11], one would end up using up to 1.5 wt% MWCNTs on average, or as used in the present study, i.e., 1.0 wt% and 2.68 wt%. The use of 2.68 wt% was mainly due to the actual calculation of the mixture.

A surfactant, Triton X-100, with a molecular weight of 625 and a critical micelle concentration (CMC) of 0.22 to 0.24 mM, was used to facilitate the dispersion of MWCNTs. When the concentration of Triton X-100 is higher than the CMC, micelles of Triton X-100 occur in the solution, and they further contribute to the dispersion quality of CNTs [19]. The concentration of Triton X-100 used in the present study was 10 times the CMC, i.e., 2.2–2.4 mM.

### 2.2. Preparation and Manufacturing

**Figure 1:**
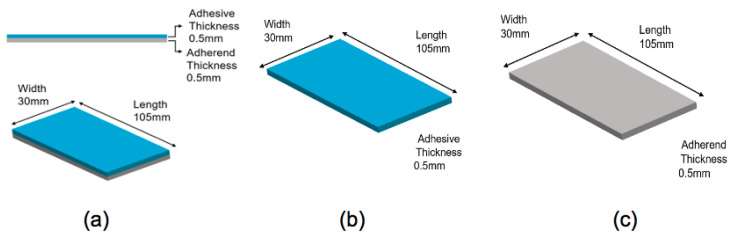
Schematics of specimens manufactured: (**a**) hybrid joint specimen; (**b**) epoxy specimen; and (**c**) aluminium specimen.

The epoxy adhesive specimens with MWCNTs and adhesive–metal bonded specimens with MWCNTs were prepared. Aluminium (Al) was used as a substrate for adhesive–metal bonded specimens. Five types of specimens were manufactured as shown in Table 1 and Figure 1.

First, the aluminium substrates were polished with 100-grit grinding paper to mechanically clean the surface from contaminants. Within an hour before bonding, 400-grit grinding paper was used to remove or minimise the thickness of the native oxide layer on the aluminium surface. Acetone was used to wipe the bonding area to thoroughly clean any residual organic contaminants on the surface, to ensure good bonding quality and the final performance of the specimens, i.e., ensuring the ultimate bond strength was reached.

**Figure 2:**
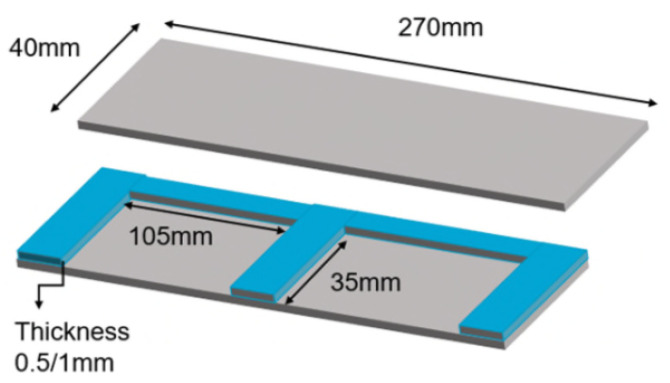
Schematic of the manufactured mould.

**Figure 3:**
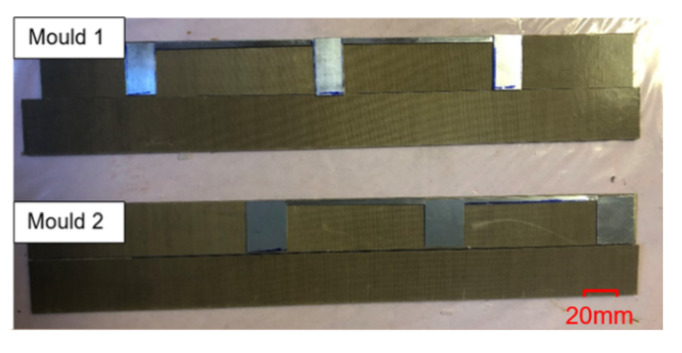
Image of the manufactured moulds.

The aluminium moulds for manufacturing the specimens were manufactured as per Figure 2 and Figure 3. The moulds were equipped with release film to avoid the residue of the polymer after curing. The surface treatment using acetone and release agent was also performed before the application of the adhesive. Mould 1 was used to manufacture specimens a and b. The thickness of the chamber in mould 1 was 1 mm; an aluminium substrate with the thickness of 0.5 mm was inserted inside to produce an adhesive thickness of 0.5 mm.

Mould 2 was used to manufacture specimens c and d; the thickness of the chamber in the mould was 0.5 mm to manufacture specimens having a thickness of 0.5 mm.

The MWCNTs were dispersed in the epoxy/adhesive system using a probe sonicator. First, the MWCNTs were weighted on a laboratory scale, and a certain weight percentage was selected relative to the weight of the epoxy. The appropriate amount of Triton X-100 was then added into the MWCNTs and 40 mL of acetone was poured into the MWCNTs and TX-100 mixture. Then the mixture was sonicated using a Branson Digital Sonifier probe sonicator for 15 min, during which the beaker was held in a water bath to prevent the solution from overheating and the acetone from evaporating.

Second, the MWCNTs’ dispersion in acetone was poured into epoxy resin, was hand-mixed to incorporate the acetone with the epoxy to lower its viscosity, and was then sonicated again for 30 min to disperse the MWCNTs into the epoxy resin. The beaker was then left to heat up under sonication energy to evaporate the acetone present in the solution.

Then, the dispersion of the MWCNTs in the epoxy was further mixed on a hot plate at 80 °C with magnetic stirring at medium speed for 5 h. This step provided full evaporation of the acetone and further dispersion of the nanotubes in the resin. The mixture was left to cool down to room temperature before a stoichiomatric ratio of hardener was added and mixed using magnetic stirring again for 15 min at medium speed. After degassing in a vacuum chamber for 30 min until no bubbles were left the mixture was applied to the mould as an adhesive using a spatula, and the mould was clamped using foldback clips. The mixture was cured at 100 °C for 5 h in a vacuum-assisted oven. The specimens were taken out from the moulds carefully after curing and were then manually trimmed with a cutter knife to remove the residuary side fillets to ensure all specimens have identical dimensions. Two specimens of each type were manufactured to demonstrate the repeatability of the results.

Two reflective strips with a length of 10 mm were attached on the surface of the specimens to allow displacement measurement using a laser extensometer. The gap between these strips was about 50 mm. A thermocouple was attached on the surface of the specimen to monitor its temperature.

### 2.3. Scanning Electron Microscopy

Scanning electron microscopy (SEM) was performed to analyse the dispersion quality of MWCNTs in the adhesive. The cross-sections of specimens a and b were analysed. The samples were cut with a scissor, and the cross-section was not polished before gold coating. A TESCAN LYRA3 SEM under secondary electron imaging mode with 10–15 kV acceleration voltage at a chamber of $10^{-3}$ mbar was used. The specimens were sputter-coated with 10 nm gold coating to minimize charging effect [20].

### 2.4. Raman Spectroscopy

Raman spectra were collected using a Raman spectrometer (Horiba LabRam 300) with a non-polarised excitation wavelength of 632.1 nm and diffraction gratings of 1800 lines/mm at 50× magnification to assess the quality of the MWCNTs [21]. The amount of defective structures and complete molecular structures were measured, and a comparison of the intensity of these two structures provided an evaluation of the quality of the MWCNTs used as the raw material in the present study. The Raman spectra of as-grown MWCNTs were collected by dispersing the MWCNTs in silicone oil and drop-casting the dispersion onto a Si substrate in order to secure the MWCNTs in place without introducing an additional fluorescence background. Raman spectra of MWCNT–adhesive composite specimens were collected from the middle section of the cross-sectional fractured edge of the specimen.

### 2.5. Thermal Strain Measurement

**Figure 4:**
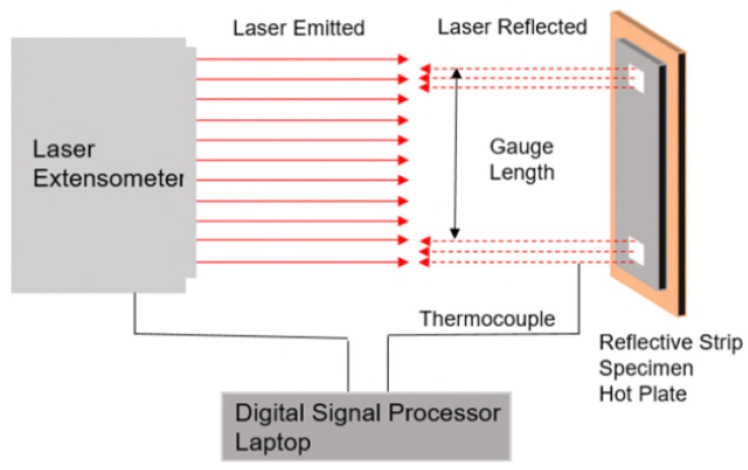
Schematic of thermal strain measurement system.

The CTE of every specimen was determined using the thermal strain measurement technique. The specimen was placed on a hot-plate and heated up to 120 °C at a rate of 5 °C/min. A Laser extensometer was used to measure the deformation of the specimen during the heating process. The schematic of the thermal strain measuring system used for the study is shown in Figure 4, and described below:

The laser extensometer was positioned at a distance of 305 mm away from the specimen, and was slightly angled (5 °C) to avoid interference from the possible spurious reflections from other reflective surfaces [22]. During the test, the laser emitted from the laser extensometer was vertically aimed at the surface of the specimen. The laser extensometer measured and indicated the gauge length between the two reflective strips according to the laser reflected from the reflective strips, and the gauge length increased with the increase of the temperature. The thermocouple monitored the temperature of the specimen during the test, and the test was stopped when the temperature reached 120 °C to avoid exceeding glass temperature. The digital signal processor transmitted the data to the computer where time and temperature data were recorded using in-house LABVIEW code. The CTE of the specimens was calculated using the following equation:

(2) $$\alpha =\frac{L_{end}-L_{start}}{L_{start}\times \left(T_{end}-T_{start}\right)}$$

where α is the CTE of the specimen, $L_{end}$ is the gauge length of the specimen at the end of the test, $L_{start}$ is the gauge length at the start of the test, $T_{end}$ is the temperature (°C) of the specimen at the end of the test, and $T_{start}$ is the temperature (°C) of the specimen at the start of the test.

## 3. Results

### 3.1. Fractography

**Figure 5:**
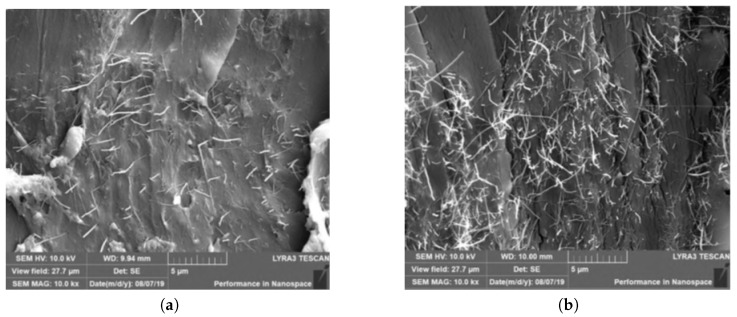
Dispersion of MWCNTs on (**a**) cross-section of specimen a with 1.0 wt% MWCNTs; (**b**) cross-section of specimen b with 2.68 wt% MWCNTs.

The SEM images in Figure 5 show the dispersion quality of the MWCNTs on the cross-section of the specimens. In Figure 5a,b, many MWCNTs protruding out from the surface of the cross-section are observed.

Cross-sectional images showed dispersion of sub-micron MWCNTs bundles within the matrices. The presence of these bundles is actually expected to occur due to the use of a high concentration of MWCNTs beyond the percolation threshold in epoxy matrices [23]. MWCNT bundles are known to be difficult to disentangle in most solvents despite of the use of surfactants [24,25]. Nonetheless, this suggests that the dispersion method used in this study is sub-optimal to fully stabilise the MWCNTs during the post-dispersion mould casting and curing process [26]. Note that the entire casting and curing process took more than 6 h to complete.

### 3.2. Raman Spectroscopy

**Figure 6:**
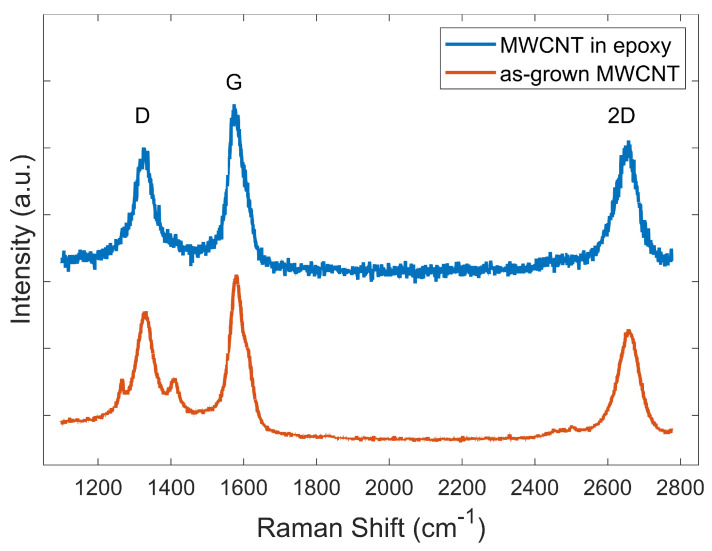
Raman spectra of MWCNTs from the cross-sectional fractured edge of composite specimens and from the as-grown MWCNTs dispersion in silicone oil.

Figure 6 shows the Raman spectrum of the MWCNTs with D and G peaks. The G band corresponds to the sp2 graphitic structure, and the D band represents the non-sp2 defects and disorder in the structure. As-grown MWCNTs used in this study exhibited a typical intensity ratio between the D and G bands, ID/IG, of 0.82, and a minor shoulder in the G band. This is typical for highly graphitic, slightly oxidised, as-grown MWCNTs synthesised by CVD using iron-containing catalysts [16,21,27]. As ID/IG < 1, these MWCNTs contained a relatively low but non-zero defect density.

Additional defects can be introduced during the MWCNTs dispersion step by the socication and high shear mixing processes. In this study, the dispersion process was carried out with reduced amplitude sonication and lower rotational speed shear mixing to minimise the introduction of additional defects.

### 3.3. Coefficient of Thermal Expansion

**Figure 7:**
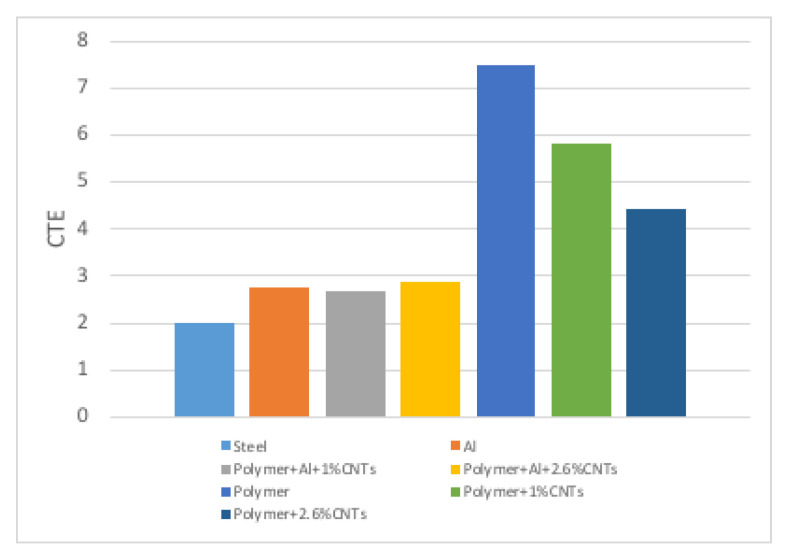
CTE ($\times 10^{-5}$ °C${}^{-1}$) comparison of different materials and specimens.

The gauge lengths of all the specimens were measured with the increase of temperature. The test started at 30 °C and ended at 120 °C. The CTEs of the specimens were calculated using Equation (2) and are listed in Table 2. The comparison of CTEs at different materials, including steel, aluminium, the polymer used in the project, and different specimens is shown in Figure 7.

Figure 7 indicates that the CTEs of hybrid joint specimen a and specimen b, which contain an aluminium substrate, were close to the CTE of the aluminium plate. This is due to the fact that the adhesive and aluminium were bonded together and the bond strength was sufficiently high that the adhesive was constrained to the expansion of the aluminium substrate during the heating process. No disbonding was observed after the tests. Additionally, due to the CTE mismatch between adhesive and aluminium, the specimens may bend. However, no bending was observed in the test as the thermal strain of the specimens was too small to be observed.

**Figure 8:**
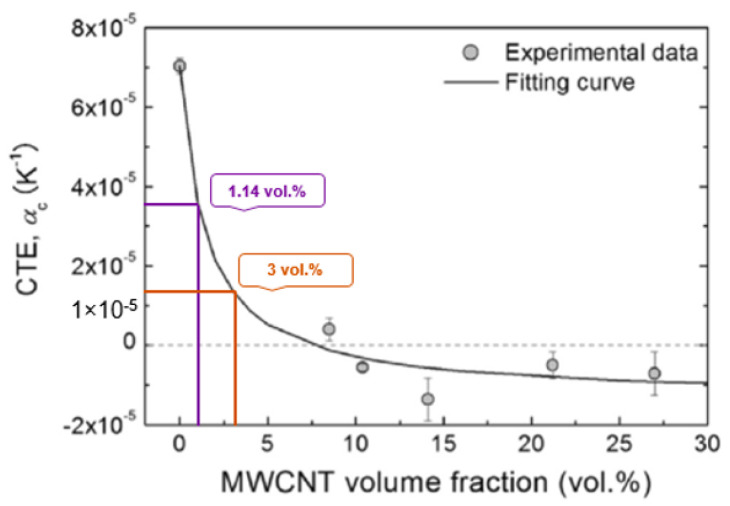
Coefficient of thermal expansion with 1.14 vol% and 3 vol% MWCNTs [11].

**Figure 9:**
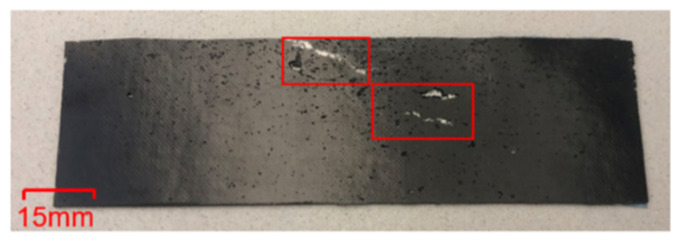
Defects on specimen: porosity (black features on the surface) and void (highlighted with red boxes).

These results are in agreement with the general trend from a previous study, see Figure 8 [11]. However, the CTE values in this study were found to be about twice as high as those approximated in the previous study at $3.6\times 10^{-5}$ °C${}^{-1}$ and $1.4\times 10^{-5}$ °C${}^{-1}$. The results in Table 2 indicate that the CTE of specimen c was $5.87\times 10^{-5}$ °C${}^{-1}$ and that of specimen d was $4.43\times 10^{-5}$ °C${}^{-1}$. The difference between the current and the previous results is attributed to the fact that:

1. Ferrocene was used as the catalyst for the CVD growth of MWCNTs and some catalysts particles might be entrapped within the MWCNTs. As a post-growth purification process was not included in this study, the weight of the catalyst might have been inadvertently included in the weight measurement of MWCNTs. Therefore, the actual weight percentages of the MWCNTs used in the specimens were most likely less than the measured weight percentages. The contribution of catalyst to the CTE of polymer is expected to be negligible [28].
2. There was a deviation in the density of MWCNTs used in the calculation to obtain volume fraction, as there was no effective method to measure the density of MWCNTs. The density of MWCNTs is related to the dimension of CNTs and the quantity of the walls in CNTs [29]. The diameter of the MWCNTs synthesised in the present study was between 20 and 100 nm. The density of the MWCNTs used in the present study might be underestimated, as the density of MWCNTs varies from 1.5 gm/cm${}^{3}$ to 2.5 gm/cm${}^{3}$ [30].
3. If the MWCNTs dispersion is unstable, re-agglomeration starts immediately after the dispersion process is stopped. CNT agglomeration induces a negative influence on the performance of polymers [23]. Agglomerates introduce inhomogeneities to the specimen that may lead to localised stress concentration under thermal load, resulting in the formation of initiation sites for failure and acceleration of breakage.
4. The defects (porosity, void etc.) in the adhesive part of the specimens may cause poor local bonding between the metal substrate and adhesive, which in turn can result in different thermal expansions of the specimens. In the present study such defects were identified; see Figure 9. Insufficient degassing of the uncured epoxy causes formation of porosity and voids.

## 4. Conclusions

The aim of the present study was to reduce the coefficient of thermal expansion (CTE) of an epoxy adhesive with the addition of multi-walled carbon nanotubes (MWCNTs) in order to reduce the CTE mismatch in adhesive–metal bonded joint systems. A low viscosity bisphenol-A epoxy resin was modified with the addition of 1 wt% and 2.68 wt% MWCNTs and was used in adhesive–metal bonded joints. The MWCNTs were synthesised by CVD and assessed by Raman spectroscopy. A significant amount of defects was observed in the MWCNTs. The dispersion quality of the MWCNTs in the epoxy was examined using scanning electron microscopy (SEM). The CTE of the adhesive–metal bonded joint was determined experimentally using the thermal strain measurement technique. The addition of 1 and 2.68 wt% of MWCNTs decreased the CTE of the polymer adhesive from $7.5\times 10^{-5}$ °C${}^{-1}$ (polymer only) to $5.87\times 10^{-5}$ °C${}^{-1}$ and $4.43\times 10^{-5}$ °C${}^{-1}$, respectively, by 22% and 41% reduction. These results are in agreement with the general trend from a previous study [11], albeit the measured CTE values in this study are about twice as high as those approximated in the previous study. The discrepancy between the experimental and the theoretical values can be attributed to the difficulties in estimating the effective density of MWCNTs and inadvertent agglomeration of the MWCNTs in the adhesive. Moreover, porosity and voids were observed on the surface of the adhesive part of the adhesive–metal bonded joints. These defects of the adhesives may result in poor bonding between the metal and the adhesive; hence the discrepancy of the experimental and the theoretical CTE.

## Figures and Tables

**Table 1:** List of specimens manufactured in the present study.

Specimen	Constitution	Dimension
Specimen a	epoxy+Al+1.00 % MWCNTs	105 mm × 30 mm × 10 mm
Specimen b	epoxy+Al+2.68 % MWCNTs	105 mm × 30 mm × 10 mm
Specimen c	epoxy+1.00% MWCNTs	105 mm × 30 mm × 5 mm
Specimen d	epoxy+2.68% MWCNTs	105 mm × 30 mm × 5 mm
Specimen e	Al only	105 mm × 30 mm × 5 mm

**Table 2:** Experimentally determined CTEs of five specimens.

Specimen	a	b	c	d	e	Polymer
Length of thermal expansion (mm)	0.127	0.133	0.257	0.218	0.132	
Interval of temperature (°C)	90	90	90	90	90	
CTE $\times \;10^{-5}$ °C${}^{-1}$	2.66	2.87	5.87	4.43	2.75	7.5
